# A2E-induced inflammation and angiogenesis in RPE cells *in vitro* are modulated by PPAR-α, -β/δ, -γ, and RXR antagonists and by norbixin

**DOI:** 10.18632/aging.203558

**Published:** 2021-09-20

**Authors:** Valérie Fontaine, Mylène Fournié, Elodie Monteiro, Thinhinane Boumedine, Christine Balducci, Louis Guibout, Mathilde Latil, José-Alain Sahel, Stanislas Veillet, Pierre J. Dilda, René Lafont, Serge Camelo

**Affiliations:** 1 Sorbonne Université, INSERM, CNRS, Institut de la Vision, Paris F-75012, France; 2 Biophytis, Sorbonne Université, Paris 75005, France; 3 Fondation Ophtalmologique Rothschild, Paris F-75019, France; 4 Department of Ophthalmology, The University of Pittsburgh School of Medicine, Pittsburgh, PA 15213, USA

**Keywords:** *N*-retinylidene-*N*-retinylethanolamine (A2E), norbixin, peroxisome proliferator-activated receptor (PPAR), retinal pigment epithelium (RPE), retinoic X receptor (RXR)

## Abstract

*N*-retinylidene-*N*-retinylethanolamine (A2E) plays a central role in age-related macular degeneration (AMD) by inducing angiogenesis and inflammation. A2E effects are mediated at least partly via the retinoic acid receptor (RAR)-α. Here we show that A2E binds and transactivates also peroxisome proliferator-activated receptors (PPAR) and retinoid X receptors (RXR). 9’-*cis*-norbixin, a di-apocarotenoid is also a ligand of these nuclear receptors (NR). Norbixin inhibits PPAR and RXR transactivation induced by A2E. Moreover, norbixin reduces protein kinase B (AKT) phosphorylation, NF-κB and AP-1 transactivation and mRNA expression of the inflammatory interleukins (IL) -6 and -8 and of vascular endothelial growth factor (VEGF) enhanced by A2E. By contrast, norbixin increases matrix metalloproteinase 9 (MMP9) and C-C motif chemokine ligand 2 (CCL2) mRNA expression in response to A2E. Selective PPAR-α, -β/δ and –γ antagonists inhibit the expression of IL-6 and IL-8 while only the antagonist of PPAR-γ inhibits the transactivation of NF-κB following A2E exposure. In addition, a cocktail of all three PPARs antagonists and also HX531, an antagonist of RXR reproduce norbixin effects on inflammation. Altogether, A2E’s deleterious biological effects could be inhibited through PPAR and RXR regulation. Moreover, the modulation of these NR by norbixin may open new avenues for the treatment of AMD.

## INTRODUCTION

AMD is the commonest cause of severe visual loss and blindness in developed countries among individuals aged 60 and older [1]. *N*-retinylidene-*N*-retinylethanolamine (A2E) is a by-product of the visual cycle [2] formed by the reaction of 2 *all-trans* retinal molecules with phosphatidylethanolamine generating phosphatidylethanolamine-bisretinoid (A2-PE), which is then hydrolysed into A2E contained in RPE [3] and Bruch’s membrane [4]. Despite the lack of spatial correlation between A2E concentration and lipofuscin autofluorescence [3], A2E has been associated with the etiology of AMD [3]. *In vitro*, A2E in presence of blue-light illumination is toxic for RPE [5–7]. Moreover, A2E itself increases the secretion of inflammatory cytokines by RPE cells *in vitro* and also in AMD animal models *in vivo* [8, 9], and the expression of VEGF *in vitro* [10] and *in vivo* [11]. However, knowledge of the mode of action of A2E for explaining these effects is limited. It has been proposed that A2E through induction of the transactivation of the α-isoform of the retinoic acid receptor (RAR) is responsible for VEGF production *in vitro* and *in vivo* [10, 11]. Indeed, pharmacological inhibition of RAR transactivation with the RAR- “specific” antagonist RO-41-5253 reduced A2E-induced VEGF production *in vitro* and *in vivo* [10, 11]. However, it has been shown that RO-41-5253 is not only a RAR antagonist, but also a partial agonist of PPAR-γ [12] suggesting that PPARs might be involved in A2E’s effects.

The RAR isoforms α, β, and γ, as well as the PPAR isoforms α, β/δ, and γ, are nuclear receptors (NR) [13]. In the presence of specific ligands, NR become activated transcription factors which bind to specific DNA regulatory elements in the promoter or vicinity of target genes leading to their transcriptional activation or repression. Many NR including RARs and PPARs form heterodimers with any of the three isoforms of RXR: RXR-α, -β or -γ [14–16]. In contrast to RAR/RXR heterodimers which are considered as non-permissive [17], for permissive heterodimers such as PPAR/RXR, the binding of an agonist of the specific NR partner or of an agonist of RXR alone is sufficient to activate the NR partner [15, 18]. Certain NRs, including RXRs and PPARs, serve as metabolic sensors containing a large hydrophobic pocket able to bind multiple ligands and are known to regulate a wide spectrum of biological pathways ranging from metabolism, cell death, inflammation and angiogenesis that are involved in AMD pathogenesis [13, 19–21]. Therefore, the role of NR in A2E’s biological effects deserves additional study.

9’-*cis*-Norbixin (norbixin) is a 6,6’-di-apocarotenoid extracted from annatto (*Bixa orellana*) seeds [22]. We have previously demonstrated that norbixin protects primary porcine RPE cells from phototoxicity induced by blue-light illumination coupled with A2E-exposure *in vitro* [23]. Moreover, norbixin reduces accumulation of A2E in RPE cells *in vitro* [23] and ocular accumulation of A2E *in vivo* [24]. Recently, it has been reported that norbixin is a PPAR-γ ligand with agonist activity [25]. However, in another cellular context it has been shown that norbixin at 20 μM did not induce PPAR-γ transactivation in 3T3-L1 adipocytes [26]. We asked how norbixin could interfere with A2E’s biological effects and if norbixin interactions with PPARs, and potentially RXRs, could be involved. To address these questions, we tested the effects of A2E in the presence or absence of norbixin on PPAR and RXR transactivation, and expression of inflammatory molecules and of VEGF in primary porcine RPE cells *in vitro*. Here, we show that A2E induces the transactivation of PPARs and RXRs. Moreover, we report that the inhibition of PPAR and RXR transactivation by norbixin is associated with a reduction of expression of VEGF, IL-6 and IL-8 and lower the transactivation of NF-κB, and AP-1, induced by A2E. The effects of norbixin are partly recapitulated by T0070907 (an antagonist of PPAR-γ), and by MK886 (an antagonist of PPAR-α) when used alone. Moreover, norbixin effects on A2E induced inflammation are fully reproduced by the antagonists of the 3 PPAR isoforms used concomitantly, as well as by a pan-antagonist of RXRs (HX531). Altogether, our results indicate that the activation of these NRs is somehow associated with A2E-induced inflammation and angiogenesis in RPE cells and can be manipulated with NR modulators including norbixin.

## RESULTS

### A2E binds and induces the transactivation of all PPAR isoforms

**Figure 1:**
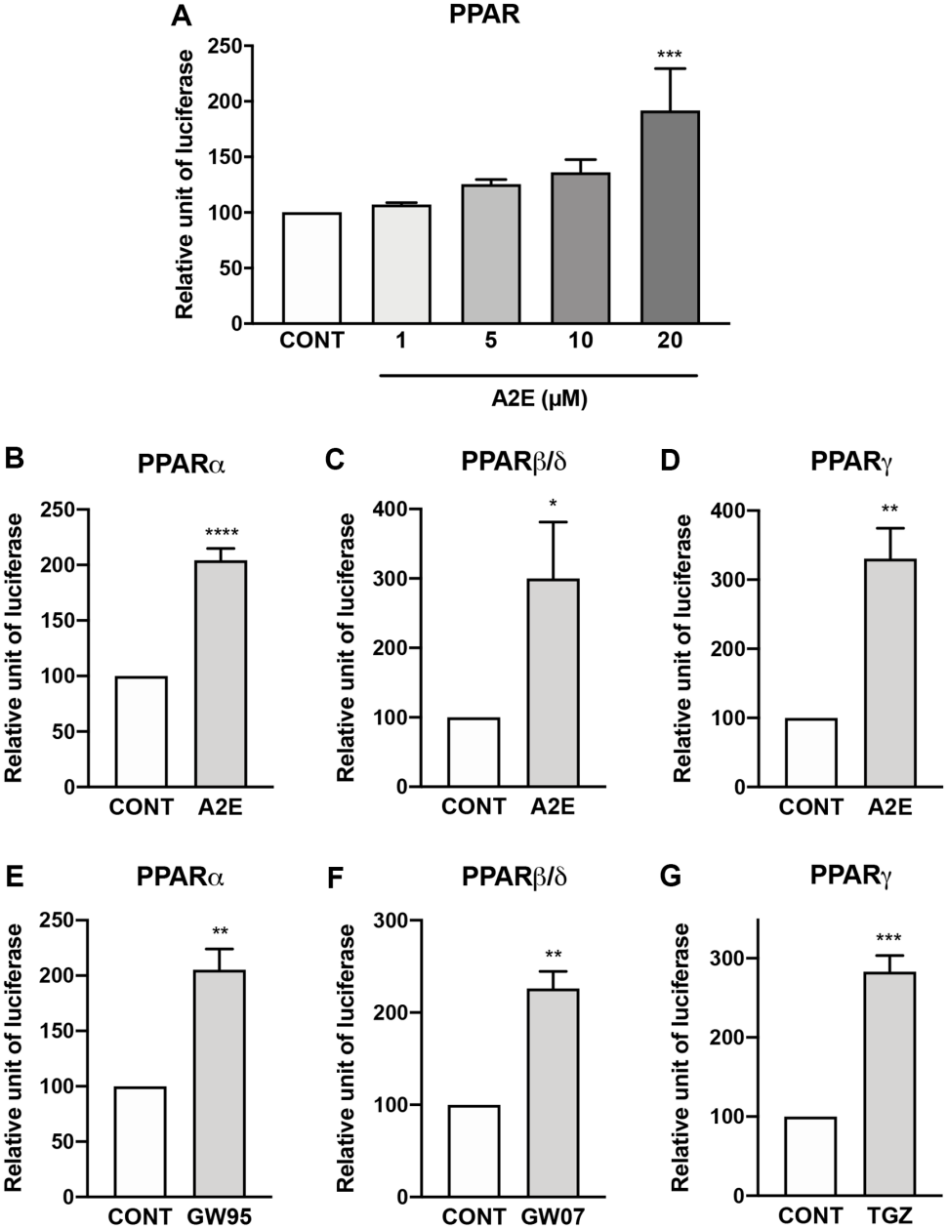
**A2E induces the transactivation of all PPAR isoforms.** Effect of increasing concentration of A2E on endogenous PPAR transactivation (**A**). Effect of A2E (20 μM) on the transactivation of over-expressed PPAR-α (**B**), PPAR β/δ (**C**) and PPAR-γ (**D**). Effect of selective PPAR agonists GW9578 (GW95, 20 μM), GW0742 (GW07, 30 μM) and troglitazone (TGZ, 20 μM) on the respective transactivation of over-expressed PPAR-α (**E**), PPAR β/δ (**F**) and PPAR-γ (**G**). Bars represent mean ± s.e.m. with *n* = 3–4. ^*^p < 0.05, ^**^p < 0.01, ^***^p < 0.001, ^****^p < 0.0001 compared to control (CONT) corresponding to DMSO alone added to the medium. (One-way ANOVA, Dunnett’s post-test).

First, we evaluated the binding profile of A2E to all three isoforms of PPAR. By competition experiments *in vitro* we determined that A2E binds to PPAR-α and -γ with low affinities (IC_50_ of 25.5 × 10^−6^ M (K_D_ = 4.1 × 10^−5^ M), and 42.7 × 10^−6^ M, (K_D_ = 1.4 × 10^−5^ M) respectively) (Table 1). Then the effects of A2E on the endogenous PPAR transactivation in porcine RPE cells were investigated by means of a luciferase assay. Dose-response experiments showed that the minimal required concentration of A2E to transactivate all PPARs in porcine RPE cells was 20 μM (Figure 1A). The level of transactivation of PPAR-α, -β/δ, or -γ isoforms by A2E was determined by co-transfection of porcine RPE cells with a plasmid expressing each of the full-length PPAR isoforms and a plasmid expressing luciferase under the control of the PPAR response element. Twenty μM of A2E induced the transactivation of the PPAR-α, -β/δ and -γ isoforms (Figure 1B, 1C, and 1D, respectively) at similar levels than much lower concentrations of their specific synthetic agonists GW9578 (Figure 1E), GW0742 (Figure 1F) and troglitazone (TGZ) (Figure 1G), respectively. Altogether our results show that despite its low affinity for PPARs, A2E is able to induce the transactivation of all three PPAR isoforms.

**Table 1:** Respective KD and IC50 for NRs of A2E and NBX.

**NR**	**K_D_ A2E**	**K_D_ NBX**	**IC_50_ A2E**	**IC_50_ NBX**
RXR-α	4.3 × 10^−6^ M	4.6 × 10^−5^ M	6.1 × 10^−6^ M	65 × 10^−6^ M
PPAR-α	4.1 × 10^−5^ M	1.5 × 10^−5^ M	42.7 × 10^−6^ M	16.5 × 10^−6^ M
PPAR-β/δ	N.T.^*^	1.5 × 10^−6^ M	N.T.^*^	9.9 × 10^−6^ M
PPAR-γ	1.4 × 10^−5^ M	1.0 × 10^−6^ M	25.5 × 10^−6^ M	1.9 × 10^−6^ M

^*^N.T.: Not tested.

### Norbixin binds to all three isoforms of PPAR, but inhibits only PPAR-γ transactivation induced by TGZ but not the transactivation of PPAR-α and -β/δ induced by high affinity agonists

**Figure 2:**
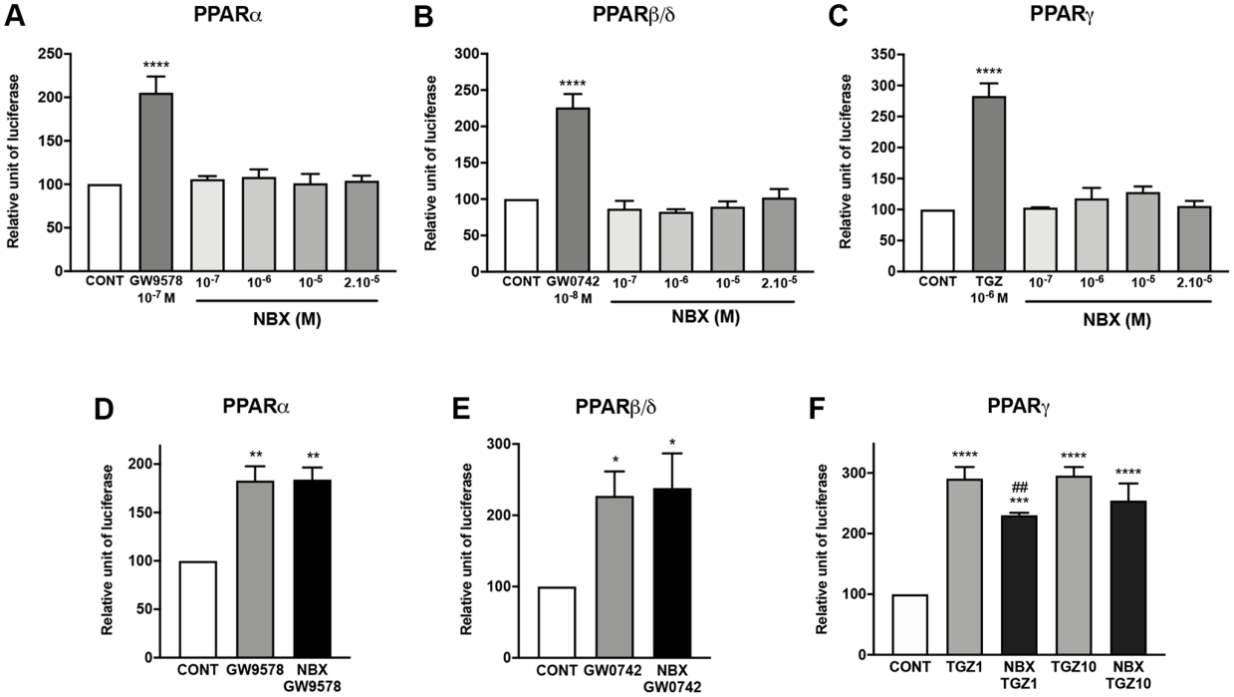
**Norbixin does not transactivate PPARs, but inhibits PPARγ transactivation induced by TGZ.** Effect of GW9578, GW0742 and TGZ and of increasing norbixin (NBX) concentrations on over-expressed PPARα (**A**), PPARβ/δ (**B**) and PPARγ (**C**). Effect of GW9578 (20 μM), of GW0742 (30 μM) and of TGZ (20 μM) alone or in competition with NBX (20 μM) on over-expressed PPARα (**D**), PPARβ/δ (**E**) and PPARγ (**F**) transactivation. Bars represent mean ± s.e.m. with *n* = 3–6. ^*^p < 0.05, ^**^p < 0.01, ^****^p < 0.0001 compared to CONT corresponding to DMSO alone added to the medium; ^#^*p* < 0.01 compared to TGZ (One-way ANOVA, Dunnett’s post-test).

The binding and activity of norbixin on PPARs remains controversial [25, 26], thus, firstly, we performed competition experiments *in vitro* between norbixin and [^3^H] GW7647, an agonist of PPAR-α, and [^3^H] rosiglitazone, an agonist of PPAR-γ. We report that norbixin binds to PPAR-α with an IC_50_ of 16.5 × 10^−6^ M and a K_D_ of 1.5 × 10^−5^ M (Table 1). However, norbixin preferentially binds to PPAR-γ (IC_50_ =1.9 × 10^−6^ M and K_D_ = 1.0 × 10^−6^ M) (Table 1). Interestingly, using an *in vitro* antagonist assay, we also observed the antagonist activity of norbixin on PPAR-β/δ with an IC_50_ of 9.9 × 10^−6^ M and an affinity of 1.5 × 10^−6^ M (Table 1). Secondly, we tested whether norbixin could induce the transactivation of PPAR-α, -β/δ or -γ in the context of RPE cells *in vitro* by co-transfecting porcine RPE cells with a plasmid expressing each of the full-length PPAR isoforms and a plasmid expressing luciferase under the control of the PPAR response element. While specific synthetic PPAR-α, -β/δ and -γ agonists (GW9578, GW0742 and TGZ, respectively) induced transactivation of the corresponding PPAR isoform (Figure 2A, 2B and 2C, respectively), norbixin did not demonstrate any transactivation activity on its own on any PPAR (Figure 2A to 2C). Then, we wanted to determine if norbixin could modulate the transactivation of PPAR isoforms induced by specific agonists. Norbixin did not inhibit significantly the transactivation of PPAR-α and -β/δ induced by their high-affinity PPAR ligands GW9578 at 10 nM (Figure 2D) and GW0742 at 1 nM (Figure 2E). Importantly, by contrast, norbixin partially inhibited PPAR-γ transactivation induced in the presence of 1 μM of TGZ (Figure 2F) but this effect was lost when TGZ was used at a higher concentration (10 μM, Figure 2F). Altogether our results show that norbixin, consistently with its low affinity for PPARs, has no inhibitory effect on the transactivation of PPAR-α and -β/δ induced by their respective synthetic high-affinity PPAR agonists. Nevertheless, norbixin is able to inhibit PPAR-γ transactivation induced by low concentrations of TGZ.

### Norbixin inhibits PPAR transactivation induced by A2E

**Figure 3:**
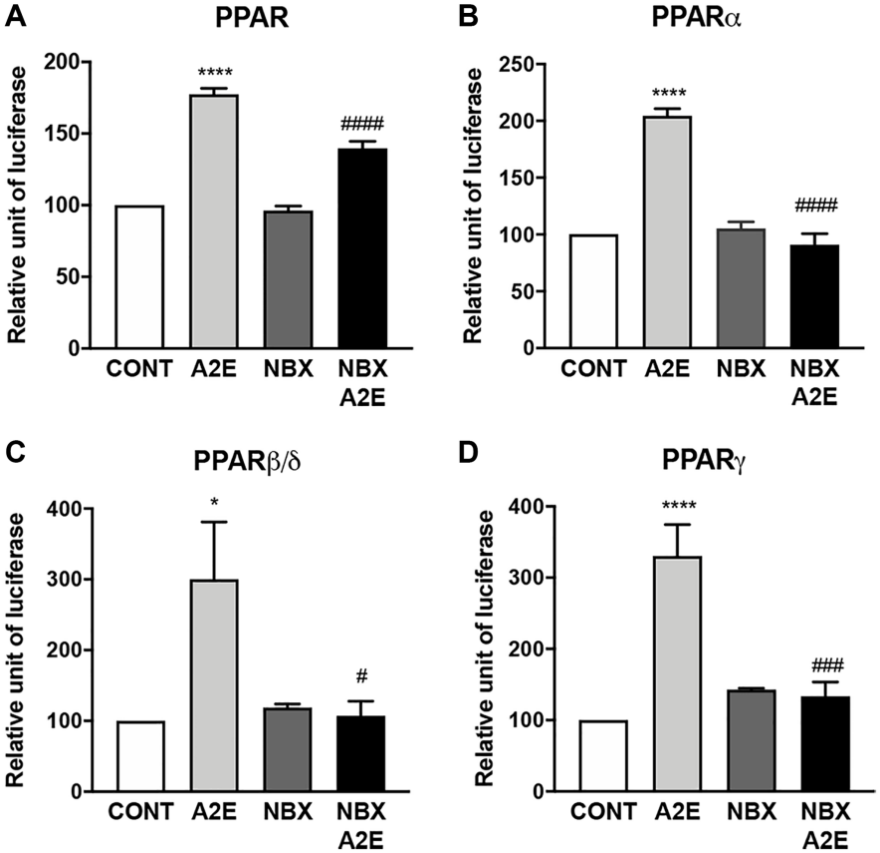
**Norbixin inhibits the transactivation of PPARs induced by A2E.** Effect of A2E (20 μM), NBX (20 μM) and A2E (20 μM) + NBX (20 μM) on endogenous PPAR transactivation (**A**). Effect of A2E (20 μM), NBX (20 μM) and A2E (20 μM) + NBX (20 μM) on over-expressed PPARα (**B**), PPARβ/δ (**C**) and PPARγ (**D**). Bars represent mean ± s.e.m. with *n* = 3–6. ^*^or ^#^*p* < 0.05, ^###^*p* < 0.001, ^****^or ^####^*p* < 0.0001 compared to CONT (DMSO alone) or to A2E, respectively (One-way ANOVA, Dunnett’s post-test).

Next, we wished to determine whether norbixin could modulate PPAR transactivation induced by A2E. We observed that norbixin significantly inhibits endogenous PPAR transactivation induced by A2E in primary porcine RPE cells by 52% (*p* < 0.0001) (Figure 3A). By contrast, treatment of RPE cells with norbixin alone did not inhibit the transactivation of endogenous PPAR. To further define if norbixin could inhibit specifically the transactivation of any of the 3 PPAR isoforms induced by A2E, we used the above-described overexpression system of the specific constructs coding for PPAR-α, β/δ or -γ co-transfected with a plasmid containing the luciferase reporter gene under the control of the PPAR response element in porcine RPE cells. As previously reported A2E induced the transactivation of all three PPAR isoforms (Figure 1 and Figures 3B to 3D) but we observed that norbixin fully abolished the transactivation of PPAR-α, PPAR-β/δ and PPAR-γ induced by A2E (Figure 3B, 3C and 3D, respectively).

In conclusion, here we show that norbixin, that does not induce nor inhibit endogenous PPAR transactivation, can inhibit PPAR transactivation induced by A2E. This indicates that norbixin behaves as a neutral antagonist of PPARs, when their transactivation is stimulated by A2E, which has a low affinity for these NR.

### Norbixin regulates the expression of inflammatory and angiogenic factors stimulated by A2E in RPE cells *in vitro*

It has been shown that A2E might play a role in AMD pathogenesis by inducing inflammation [8, 9] and neovascularization through the enhanced expression of VEGF *in vitro* and *in vivo* [10, 11]. PPARs could be involved in these processes [13]. We have shown that norbixin partially inhibits endogenous PPAR transactivation induced by A2E. Therefore, we aimed to decipher the effects of norbixin on A2E-induced expression of molecules involved in inflammation and angiogenesis in RPE cells *in vitro*.

**Figure 4:**
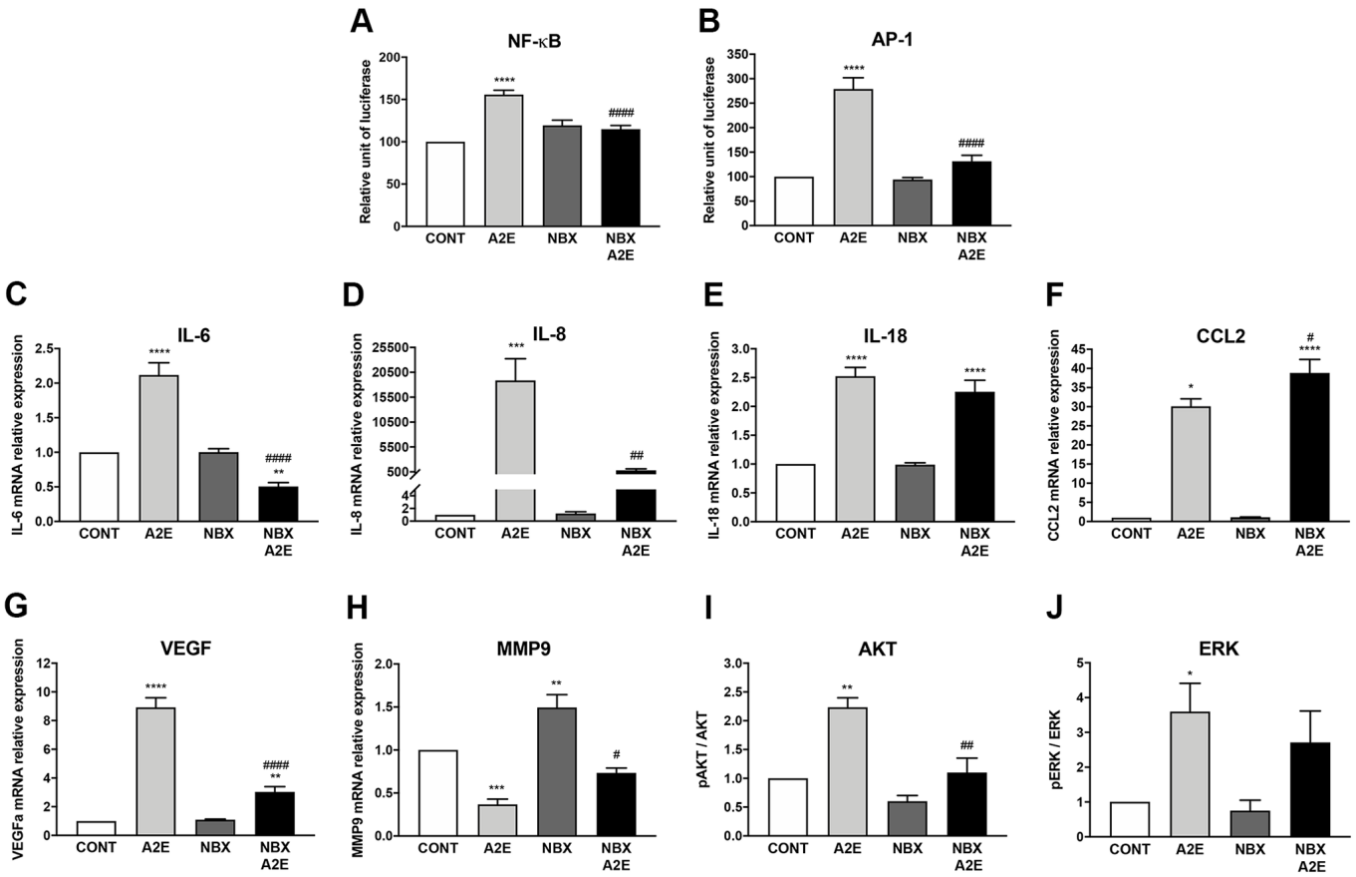
**Norbixin inhibits the transactivation of NF-κB and AP-1 and regulates the expression of inflammatory and angiogenic factors stimulated by A2E in RPE cells *in vitro*.** Effect of A2E (20 μM), NBX (20 μM) and NBX (20 μM) + A2E (20 μM) on NF-κB (**A**) and AP-1 (**B**) transactivation. Effect of A2E (30 μM), NBX (20 μM) and NBX (20 μM) + A2E (30 μM) on IL-6 (**C**), IL-8 (**D**), IL-18 (**E**), CCL2 (**F**), VEGF (**G**) and MMP9 (**H**) mRNA expression, and on AKT (**I**) and ERK (**J**) protein phosphorylation. Bars represent mean ± s.e.m. with *n* = 3–9. ^*^or ^#^*p* < 0.05, ^**^or ^##^*p* < 0.01, ^***^p < 0.001, ^****^or ^####^*p* < 0.0001 compared to CONT (DMSO alone) or to A2E, respectively (One-way ANOVA, Dunnett’s post-test).

Firstly, we compared the transactivation of transcription factors involved in inflammation regulation and the expression of inflammatory cytokines induced by A2E in the presence or absence of norbixin. NF-κB transactivation induced by A2E was strongly downregulated by norbixin (by 73%, *p* < 0.0001, Figure 4A). A2E also induced transactivation of AP-1, a key modulator in inflammatory cytokine expression, which was also inhibited by norbixin (82%, *p* < 0.0001, Figure 4B). Accordingly, norbixin was able to significantly reduce (by 145%, *p* < 0.0001) the mRNA expression of IL-6 induced by A2E (Figure 4C). Following norbixin treatment, the level of IL-6 was even lower than the basal level found in RPE cells not treated with A2E. We observed that norbixin strongly inhibited (by approximately 23-fold, *p* < 0.01) IL-8 mRNA expression as well (Figure 4D). It has been previously shown that A2E activates the NLRP3 inflammasome complex [8]. In our *in vitro* model we observed that mRNA expression of IL-18, a cytokine of the IL-1 family, depending on NLRP3 activation, and that has been implicated in AMD pathogenesis [27], was induced by A2E in RPE cells (Figure 4E). In contrast with IL-6 and IL-8, IL-18 expression was not inhibited in the presence of norbixin (Figure 4E). The expression of the chemokine CCL2, that is also implicated during AMD through macrophage recruitment in the subretinal space [28], was up-regulated by A2E (Figure 4F). Unexpectedly, its expression was not inhibited but was further increased by norbixin treatment (Figure 4F). Altogether, norbixin displays differential modulatory effects on the expression of pro-inflammatory molecules.

Secondly, we investigated angiogenesis, the major driver of neovascular AMD, during which VEGF promotes the growth of neo-vessels originating from the choriocapillaris towards the retina. In our *in vitro* model of porcine RPE cells, we confirmed that A2E stimulation enhanced significantly (by approximately 9-fold) the mRNA expression of VEGF (Figure 4G). Interestingly, this increase in VEGF expression was significantly inhibited by addition of norbixin (Figure 4G). VEGF alone is not sufficient to induce neovascular AMD if Bruch’s membrane remains intact [29]. Matrix metalloproteinases and in particular MMP9 are able to alter Bruch’s membrane integrity [29], thus potentially allowing neo-vessels responsible for blindness in neovascular AMD to reach the neural retina. Since MMP9 production has been shown to be under the control of PPAR-α and -γ [30], we measured MMP9 mRNA expression in our *in vitro* model of porcine RPE cells stimulated by A2E in presence or in absence of norbixin. A2E reduced the expression of MMP9 mRNA (Figure 4H), whereas norbixin slightly, but significantly, increased MMP9 expression in the absence or presence of A2E (Figure 4H).

Finally, it has been shown that AKT and ERK inhibition suppress IL-6, IL-8 and VEGF production in RPE cells stimulated by 7-ketocholesterol [31]. Thus, we reasoned that, since A2E also induces IL-6, IL-8 and VEGF production, ERK and AKT could also be targets of A2E and that their action could be regulated by norbixin. We observed that A2E indeed induced the phosphorylation of AKT (Figure 4I) and of ERK (Figure 4J) in primary porcine RPE cells. Treatment with norbixin abrogated completely the increase of AKT phosphorylation (Figure 4I). By contrast, despite a small trend towards reduction, ERK phosphorylation was not significantly inhibited by norbixin (Figure 4J). Altogether, our observations indicate that norbixin modulates inflammation and angiogenesis induced by A2E in RPE cells *in vitro*. We make the hypothesis that this modulation could be related to norbixin inhibitory effect on PPAR transactivation induced by A2E.

### PPAR-α and PPAR-γ antagonists partly reproduce the effects of norbixin on inflammation, but not on VEGF expression induced by A2E in RPE cells *in vitro*

**Figure 5:**
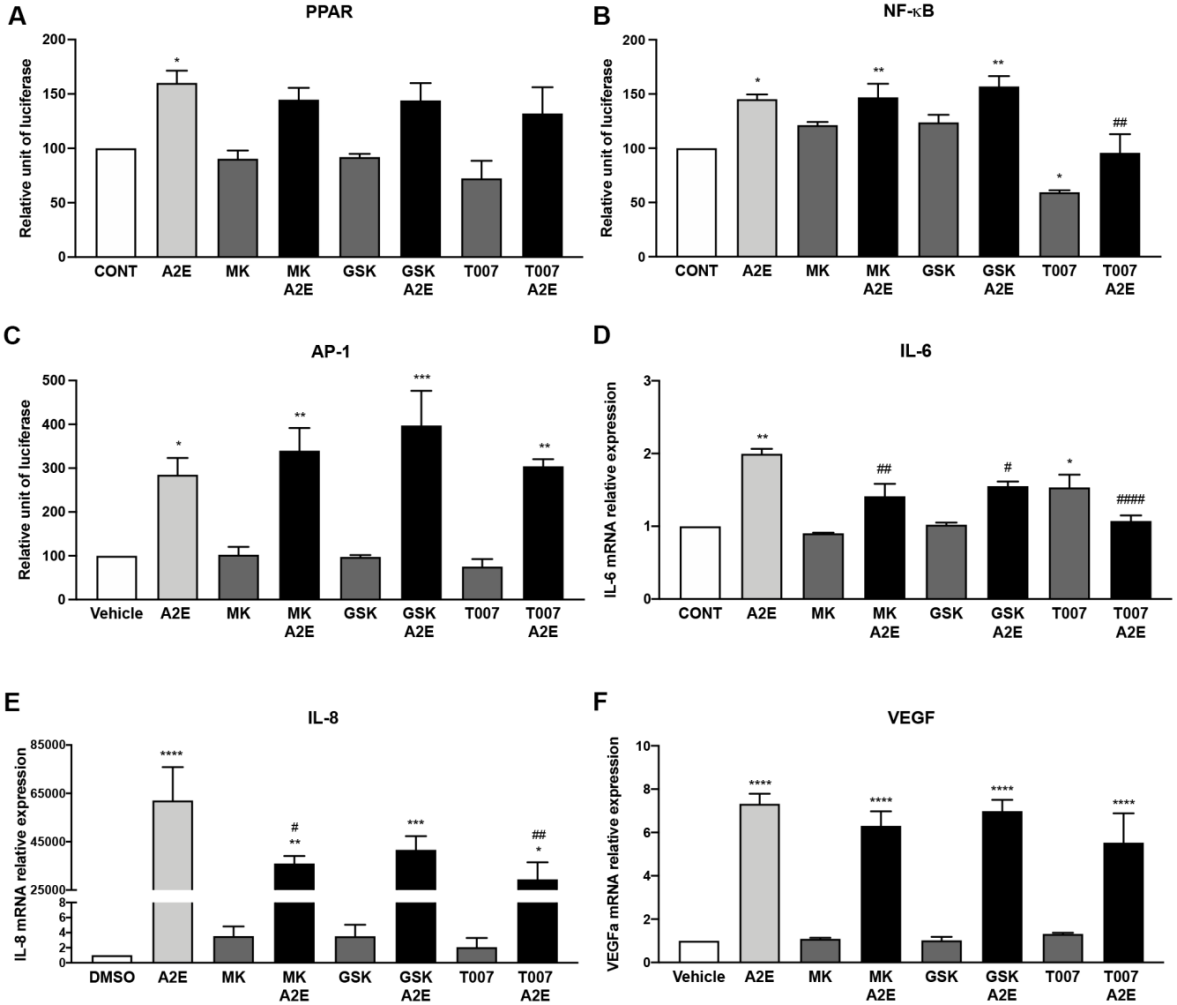
**PPAR-α and PPAR-γ antagonists partly reproduce the effects of norbixin on inflammation but not on VEGF expression induced by A2E in RPE cells *in vitro*.** Effect of PPAR-α, -β/δ and -γ selective antagonists MK886 (MK, 1 μM), GSK3787 (GSK, 1 μM) and T007907 (T007, 10 μM) respectively alone or with A2E (20 μM) on PPAR (**A**), NF-κB (**B**) and AP-1 (**C**) transactivation, and on IL-6 (**D**), IL-8 (**E**) and VEGF (**F**) mRNA expression. Bars represent mean ± s.e.m. with *n* = 3 ^*^ or ^#^*p* < 0.05, ^**^ or ^##^*p* < 0.01, ^***^p < 0.001, ^****^ or ^####^*p* < 0.0001 compared to CONT (DMSO alone) or to A2E, respectively (One-way ANOVA, Dunnett's post-test).

To test this hypothesis, we measured the action of the specific antagonists of PPAR-α (MK886), β/δ (GSK3787) and γ (T007907) on inflammation and angiogenesis induced by A2E in RPE cells *in vitro*. We determined the effects of the PPAR isoform antagonists on A2E-induced PPAR, NF-κB, and AP-1 transactivation, on the expression of the inflammatory cytokines (IL-6, IL-8) and of VEGF that were clearly inhibited by norbixin treatment (Figure 4). The PPAR-α (MK886), β/δ (GSK3787) and γ (T007907) antagonists, taken separately, did not significantly inhibit the transactivation of PPAR induced by A2E (Figure 5A). Nevertheless, the endogenous levels of NF-κB transactivation was inhibited by T007907 (*p* < 0.05, Figure 5B). Moreover, we observed that NF-κB transactivation induced by A2E were entirely inhibited in presence of T007907 (100 %, *p* < 0.01), a PPAR-γ specific antagonist, but not by PPAR-α or β/δ antagonists (Figure 5B). By contrast, none of the PPAR isoform antagonists had any effect on the transactivation of AP-1 (Figure 5C). When we measured the expression of the inflammatory cytokines IL-6, IL-8 and of VEGF, we noted that treatment with MK886, GSK3787 and T007907 reduced significantly IL-6 expression induced by A2E (by 58.7%, 45% and 69.6% respectively, Figure 5D). Furthermore, IL-8 expression induced by A2E was also inhibited by MK886, GSK3787 and T007907 (by 37%, 35% and 45.9%, respectively, Figure 5E). Finally, we observed that none of the PPAR isoform antagonists alone had any effect on VEGF expression induced by A2E in RPE cells *in vitro* (Figure 5F). In conclusion, while none of the PPAR selective antagonists used alone reproduced all of norbixin’s effects and modulated VEGF expression, these experiments point to an important role of PPAR in the regulation of inflammation induced by A2E in RPE cells *in vitro*.

### A cocktail of three PPAR antagonists reproduces most of the effects of norbixin on inflammation observed in RPE cells *in vitro*

**Figure 6:**
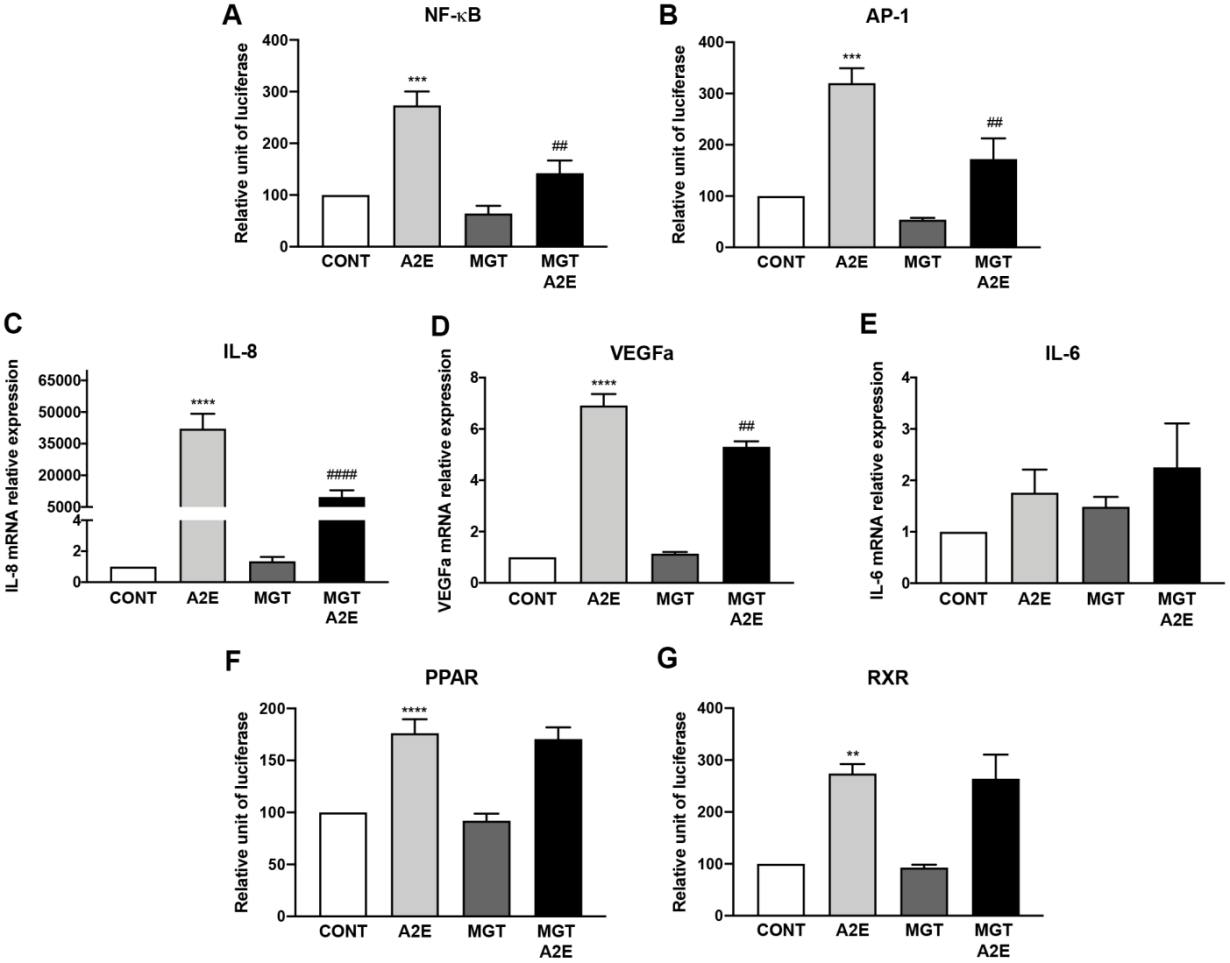
**PPAR-α, PPAR- β/δ and PPAR-γ antagonists used in co-treatment partly reproduce the effects of norbixin on inflammation but not on IL-6 expression and PPAR transactivation induced by A2E in RPE cells *in vitro*.** Effect of a mixture of PPAR-α, -β/δ and -γ selective antagonists MK886 (MK, 1 μM), GSK3787 (GSK, 1 μM) and T007907 (T007, 10 μM) alone or with A2E (20 μM) on NF-κB (**A**), AP-1 (**B**) transactivation, and on IL-8 (**C**), VEGFa (**D**) and IL-6 (**E**), mRNA expression. Effects on PPAR (**F**) and RXR (**G**) transactivation. Bars represent mean ± s.e.m. with *n* = 3–6 and ^**^ or ^##^*p* < 0.01, ^***^p < 0.001, ^****^ or ^####^*p* < 0.0001 compared to CONT (DMSO alone) or to A2E, respectively (One-way ANOVA, Dunnett's post-test).

Since none of the PPAR-α, β/δ and γ antagonists applied alone could reproduce fully the effect of norbixin that inhibits the transactivation of all three PPARs simultaneously we wished to test the effects of the specific PPAR antagonists (MK886, GSK3787, and T007907) on inflammation and angiogenesis induced by A2E in RPE cells *in vitro*. We observed that this cocktail of antagonists was able to inhibit NF-κB (*p* < 0.01, Figure 6A), and AP-1 (*p* < 0.01, Figure 6B) transactivation, and the expression of IL-8 (*p* < 0.0001, Figure 6C) and of VEGF (*p* < 0.01, Figure 6D) but not of IL-6 (*p* > 0.05, Figure 6E). In order to definitively associate PPAR transactivation and inflammation induced by A2E we wanted to verify the effect of this cocktail of PPAR antagonists on PPAR. Surprisingly the combination of the three PPAR antagonists was not able to reduce PPAR transactivation (Figure 6F) in our *in vitro* test. Due to the permissive nature of PPAR, we hypothesized that A2E could induce PPAR transactivation through binding on RXR homodimers. Therefore, we tested the effects of the combination of the three PPAR antagonists on RXR transactivation induced by A2E but no effects were observed (Figure 6G). Altogether, our results suggest that inhibition of the three PPAR isoforms is necessary to reproduce the effects of norbixin in the regulation of inflammation induced by A2E in RPE cells *in vitro*. However, inhibition of PPAR transactivation could only be observed if RXR transactivation is inhibited concomitantly.

### Norbixin binds to RXR-α and inhibits the transactivation of RXR induced by A2E

**Figure 7:**
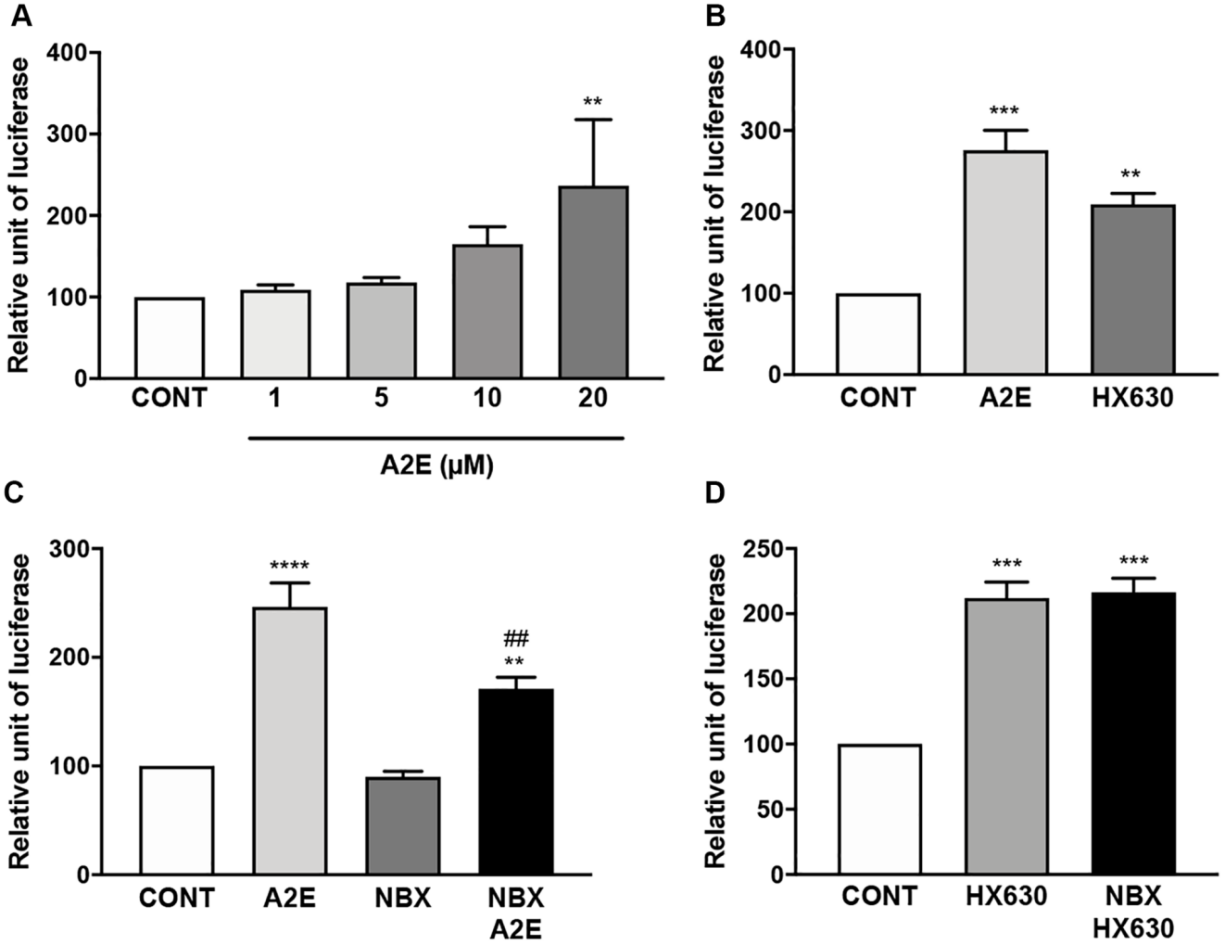
**Norbixin inhibits the transactivation of RXRs induced by A2E.** Effect of increasing concentrations of A2E on RXR transactivation (**A**). Effect of A2E (20 μM) and HX630 (5 μM) on RXR transactivation (**B**). Effect of A2E (20 μM), NBX (20 μM) and A2E (20 μM) + NBX (20 μM) on RXR transactivation (**C**). Effect of HX630 (5 μM) alone or in competition with NBX (20 μM) on RXR transactivation (**D**). Bars represent mean ± s.e.m. with *n* = 3–5. ^**^ or ^##^*p* < 0.01, ^***^p < 0.001, ^****^p < 0.0001 compared to CONT (DMSO alone) or to A2E, respectively (One-way ANOVA, Dunnett's post-test).

Based on the above hypothesis, since norbixin inhibits inflammation and angiogenesis as well as PPAR transactivation induced by A2E this suggest that norbixin could also modulate RXR transactivation. To confirm this hypothesis, we tested by competition experiments whether A2E and norbixin could bind to RXR-α. We observed that A2E is a ligand of RXR-α with an IC_50_ of 6.1 × 10^−6^ M (Table 1). In a similar experiment by competition *in vitro* between norbixin and [^3^H]9-*cis*-retinoic acid we observed that norbixin also binds to RXR-α, but with 10-times lower affinity (IC_50_ of 65 × 10^−6^ M and K_D_ of 4.6 × 10^−5^ M) (Table 1). We confirm that A2E induces the transactivation of endogenous RXRs in porcine RPE cells (Figure 7A) at a minimal concentration of 20 μM. Transactivation levels of RXRs by A2E are equivalent to the transactivation induced by HX630, a specific RXR agonist, at 5 μM (Figure 7B). In similar transactivation experiments, we tested the activity of norbixin alone or added to A2E on RXR transactivation. Norbixin alone did not induce the transactivation of RXRs (Figure 7C). However, norbixin inhibited significantly (by 49%, *p* < 0.01) the transactivation of RXRs induced by A2E in porcine RPE cells *in vitro* (Figure 7C). By contrast, norbixin did not inhibit the transactivation of RXRs induced by the specific agonist HX630 (Figure 7D). Based on these observations it could be hypothesized that the modulation by norbixin of RXR transactivation induced by A2E might be involved in the inhibition of inflammation and angiogenesis induced by A2E in RPE cells *in vitro*.

### Partial pharmacological inhibition of RXR and PPAR transactivation by the pan-RXR antagonist HX531 reduces A2E-induced inflammation and angiogenesis

**Figure 8:**
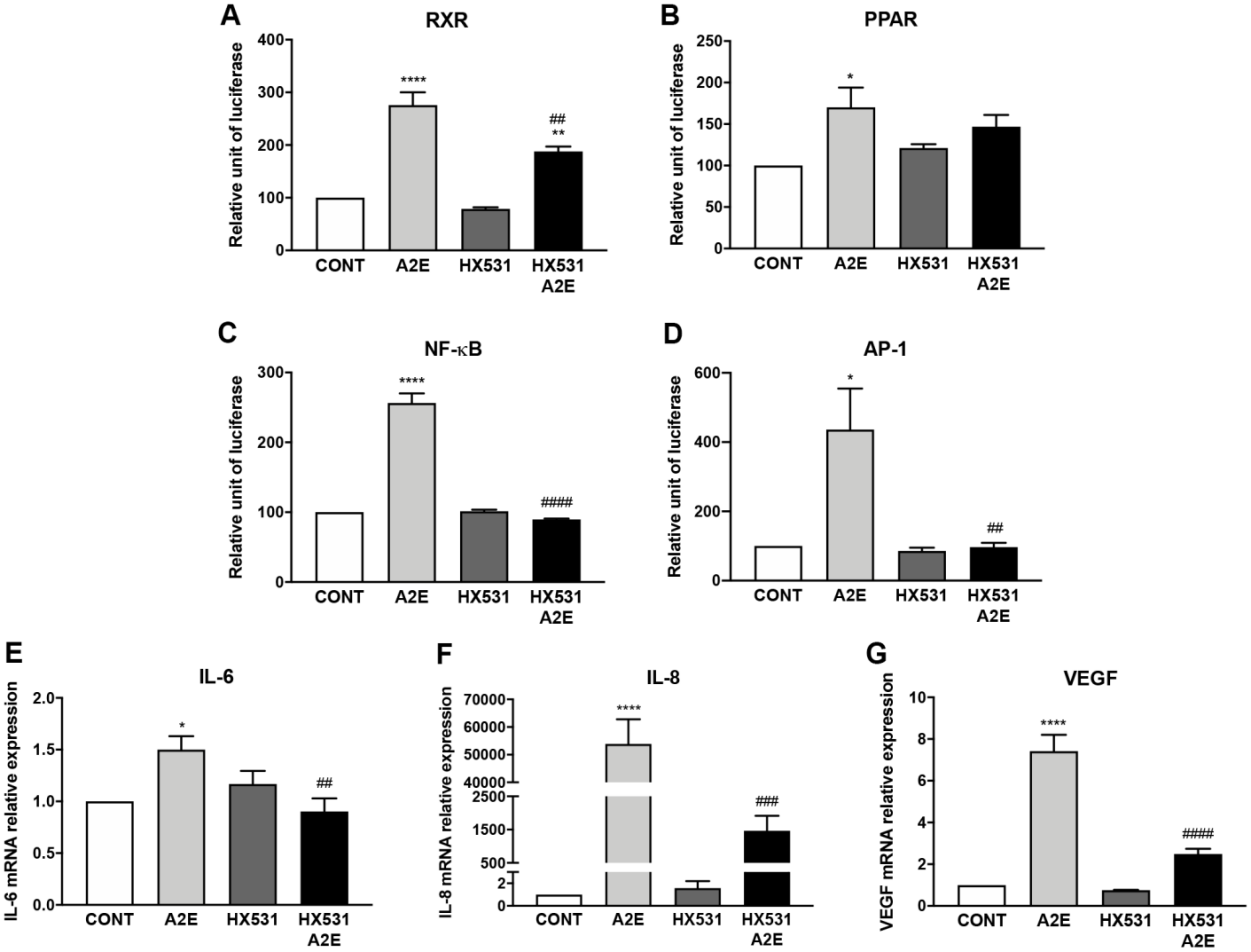
**Partial inhibition of RXR and PPAR transactivation by the pan-RXR-antagonist HX531 reduces A2E-induced inflammation and angiogenesis.** Effect of A2E (20 μM), HX531 (5 μM) alone and HX531 (5 μM) + A2E (20 μM) on RXR (**A**), PPAR (**B**), NF-κB (**C**) and AP-1 (**D**) transactivation, and on IL-6 (**E**), IL-8 (**F**) and VEGF (**G**) mRNA expression. Bars represent mean ± s.e.m. with *n* = 3–4. ^*^p < 0.05, ^**^ or ^##^*p* < 0.01, ^###^*p* < 0.001, ^****^ or ^####^*p* < 0.0001 compared to CONT (DMSO alone) or to A2E respectively (One-way ANOVA, Dunnett's post-test).

As we hypothesized that norbixin’s effects on inflammation and angiogenesis could be associated with a partial inhibition of RXR transactivation, we tested whether a synthetic RXR pan-antagonist (HX531) would reproduce these effects. Firstly, we confirmed that HX531 partially interferes with the transactivation of RXR induced by A2E (50.1% inhibition, *p* < 0.01; Figure 8A). We also observed that HX531 tended to partially inhibit PPAR transactivation but this effect was not significant (30%; ns. Figure 8B). We then measured the transactivation of NF-κB and AP-1 induced by A2E in presence or absence of HX531. A2E- induced transactivation of NF-κB, was fully downregulated by HX531 (100% inhibition, *p* < 0.0001, Figure 8C). Similarly, HX531 entirely inhibited the transactivation of AP-1 induced by A2E (100%, *p* < 0.0001, Figure 8D). Both NF-κB and AP-1 are transcription factors critically involved in inflammation regulation. Accordingly, we observed that HX531 strongly and significantly reduced (by 88.6%, *p* < 0.01) the mRNA expression of IL-6 induced by A2E (Figure 8E). HX531 also drastically inhibited IL-8 mRNA expression induced by A2E in RPE cells *in vitro* (99.6%, *p* < 0.001, Figure 8F). In addition, mRNA expression of VEGF was strongly lowered by treatment with HX531 (76.8%, *p* < 0.0001, Figure 8G). Altogether, these observations seem to confirm that modulation of VEGF and inflammatory cytokines, expression by norbixin may be associated with partial inhibition of RXR as well as PPAR transactivation.

## DISCUSSION

A2E, a phototoxic by-product of the visual cycle [2], has been associated with the etiology of AMD despite the lack of spatial correlation between A2E and fundus autofluorescence [3]. Indeed, A2E increases the secretion of inflammatory cytokines [8, 9] and of VEGF by RPE cells *in vitro* and also in AMD animal models *in vivo* [10, 11]. In the present study, we confirm and extend these previous observations by showing that A2E induces the transactivation of NF-κB, and AP-1, the phosphorylation of AKT and ERK, and induces the expression of IL-6, IL-8, IL-18, CCL2, and VEGF. By contrast A2E reduces the expression of MMP9 by porcine RPE cells *in vitro*.

### Effects of A2E on NRs

Albeit A2E is a ligand of RAR-α and that it has been proposed that most of A2E’s effects in RPE cells *in vitro* are mediated through RAR transactivation [10, 11], the mechanism of action of A2E remains poorly described. Here, we show that A2E binds to, and induces the transactivation of all three isoforms of PPAR in RPE cells *in vitro*. We are not the first to point out the potential importance of NRs, and particularly PPARs, in controlling several AMD-pathogenic pathways [32–37]. Moreover, our results indicate that the three PPAR isoforms may play different roles during AMD. Accordingly, while PPAR-α agonists have been shown to protect RPE cells against an oxidative stress *in vitro* [38] and to reduce angiogenesis in mouse models of neovascular AMD [39], pharmacological inhibition of PPAR-β/δ limited angiogenesis, but exacerbated signs of dry AMD [40]. Conversely, activation of PPAR-β/δ reduced lipid accumulation in RPE cells *in vitro*, a symptom of intermediate dry AMD [40]. Additionally, the PPAR-γ agonist TGZ protected RPE cells from an oxidative stress insult [41], whereas other PPAR-γ agonists (pioglitazone and rosiglitazone) exacerbated cell death induced by *t*-butyl hydroperoxide treatment [41]. Moreover, PPAR-γ activation by TGZ reduced VEGF production in RPE cells, inhibited tube formation in choriocapillaris endothelial cells *in vitro* and limited angiogenesis in a rat model of CNV [42]. Our results confirm the important, but versatile, role of PPAR isoforms during AMD.

We tempted to determine the exact role of each PPAR isoform in the modulation of inflammatory and angiogenesis markers using PPAR-α- (MK886), PPAR-β/δ- (GSK3787) and PPAR-γ- (T0070907) specific antagonists, None of the PPAR antagonists alone inhibited VEGF expression and AP-1 transactivation induced by A2E. By contrast, our results demonstrate that IL-6 and IL-8 expression induced by A2E were inhibited by any PPAR antagonists. Interestingly, T0070907, a selective antagonist of PPAR-γ, also inhibited NF-κB transactivation with or without A2E but surprisingly enhanced endogenous levels of IL-6 mRNA expression suggesting that PPAR-γ plays a complex role in regulation of inflammation in RPE cells. This suggests that PPAR antagonisms, at least indirectly, inhibit inflammation induced by A2E in our cellular model. To strengthen this point we performed similar experiments this time inhibiting the transactivation of all three PPARs concomitantly using a mix of PPAR-α, β/δ and γ antagonists, and we showed that in this case the expression of all inflammatory molecules (with the exception of IL-6) and the activation of AP-1 and NF-kB were inhibited. This observation reproduces the effects on inflammation, of norbixin that inhibits the transactivation of all three PPARs simultaneously. However, surprisingly the use of the three PPAR antagonists together could not significantly reduce PPAR transactivation. Due to the permissive nature of the PPAR which can be activated by an agonist of RXRs alone [15, 18], we propose that PPAR transactivation observed in these experimental settings could be induced indirectly by the binding of A2E on RXRs. In this case however, we hypothesize that the transactivation of PPARs in presence of the three PPAR antagonists would be suboptimal and would not allow for the induction of inflammation. From our present study, however, we cannot affirm that the transactivation of PPARs induced by A2E is directly responsible for inflammatory marker expression. Further experiments using either PPAR-α, β/δ and γ agonists alone or in combination would be required to determine the exact role of PPAR transactivation on cytokine production by RPE cells.

PPAR mediated regulation of inflammation and angiogenesis in response to A2E in the context of AMD is certainly complex. Indeed, PPARs, as said above, can form homodimers, or associate with RXRs to form permissive PPAR/RXR heterodimers [15, 18]. Direct ligand interaction to PPARs or RXRs could result in binding of PPAR/RXR heterodimers on the PPAR response elements and modulation of gene expression [15, 18]. As said above RXR/RXR homodimers [15, 18] can also directly bind to the PPAR response elements [43]. We report here that RXRs are also essential in A2E’s effects in RPE cells *in vitro*. We determined the affinity of A2E for RXR-α and we showed that A2E induces the transactivation of RXRs. Moreover, partial pharmacological inhibition of RXR transactivation by HX531 reduced drastically the expression of IL-6, IL-8, and of VEGF and reduced the transactivation of NF-κB and AP-1.

Importantly, the fact that A2E induces RXR transactivation, the major NR heterodimer partner, implies that A2E may also induce the transactivation of other permissive NRs such as LXRs which have recently been reported to play an important role in AMD physiopathology [44]. It has been demonstrated that LXRs regulate, at least partially, the expression of VEGF [45] and MMP9 [46]. Moreover, LXRs have been shown to modulate the expression of APOE and ABCA1 [44] that are important for retinal homeostasis by controlling the efflux of cholesterol internalized during daily uptake of photoreceptor cells tips. The precise effects of A2E on LXR transactivation will be the subject of future experiments.

The regulation of inflammatory marker expression by A2E might implicate other non-permissive NRs such as RARs as previously described in the case of VEGF [10]. As an example of RAR involvement, it has been shown that 9-*cis*-RA, a bi-specific agonist of RARs and of RXRs induces CCL2 expression in THP-1 cells, a human monocytic cell line [47]. Thus, the transactivation of RARs [10] and RXRs by A2E could explain the induction of CCL2 by A2E in our model. Further experiments are required to further decipher the role of RARs during AMD. Other NRs might also be involved in regulation of IL-18 expression and ERK phosphorylation that were induced by A2E. The identification of these putative NRs and of their role also deserve further studies.

Nevertheless, our results are novel and important, because they allow a better understanding of A2E potential mode(s) of action and implication in AMD pathogenesis. We demonstrate that A2E, which plays a central role in AMD, behaves as a weak (or medium) affinity PPAR pan-agonist as well as an RXR agonist. Moreover, we report that, in RPE cells *in vitro*, A2E’s effects on cytokines regulation and angiogenesis, are controlled at least partly through RXR and PPAR modulation. Altogether, we cannot affirm that there is a direct link between PPAR activation by A2E and cytokine production but we can stress out that inhibition of PPAR and/or RXR transactivation is at least an indirect way to modulate inflammation.

As previously reported, A2E activates NLRP3 a central transcription factor regulating the expression of certain inflammatory cytokines including IL-1 and IL-18 [8]. In our culture system the level of IL-1 produced was below detection limits. By contrast, high levels of IL-18 production were induced by A2E. Activation of NLRP3 could be interpreted as a danger signal due to the toxic effects of A2E. However, in the present experiments IL-18 production was not dampened by treatment with norbixin or any of the PPAR antagonists. This could suggest that the toxicity of A2E responsible for NLRP3 activation and IL-18 production is not regulated through NRs. Moreover, in our cell culture conditions, in absence of blue light exposure used for the dosage of cytokine production, cell viability was not diminished by A2E. Therefore, in our experiments, the toxic effect of A2E and its capacity to promote inflammatory cytokine production seem to be independent phenomena.

### Effects of norbixin on inflammation and A2E-induced NR transactivation

The antioxidant formulations AREDS (Age-Related Eye Disease Studies) and AREDS2 containing the carotenoids β-carotene, or lutein and zeaxanthin are given to patients with early stages of AMD to slow down the evolution of the disease [48, 49]. Interestingly, β-carotene and lutein/zeaxanthin have been shown to interact with or modulate the expression of PPARs and/or RXRs [50–52]. Exploration of the role of PPARs and RXRs in carotenoid protective effects may help improve their clinical efficacy. Here we tested the effects of norbixin a di-apocarotenoid that protects primary porcine RPE cells against A2E and blue-light exposure *in vitro* and is protective in various *in vivo* animal models of AMD [23, 24]. In agreement with a previous study showing that norbixin reduced NF-κB and IL-6 expression *in vivo* [25], here we demonstrated the modulatory effects of norbixin on A2E-induced expression of IL-6, IL-8, MMP9, CCL2 and VEGF, as well as on the transactivation of NF-κB and of AP-1, and on the phosphorylation of AKT (Figure 8). These observations led us to hypothesize that the interference of norbixin with A2E biological effects could be due to direct interactions between norbixin and PPARs and/or RXRs.

Based on *in silico* and *in vivo* studies it had been previously reported that norbixin was a PPAR-γ agonist like pioglitazone [25]. However, as NR responsiveness to ligand binding depends on the specific cellular context and experimental settings [15, 18], it has also been shown that norbixin at 20 μM does not induce PPAR-γ transactivation in 3T3-L1 adipocytes, but induces a limited transactivation of PPAR-γ at 70 μM [26]. However, in the fresh porcine RPE cells used in the present study and that is central in AMD, norbixin did not induce the transactivation of any of the PPARs (α, β/δ and γ) at any concentration used as demonstrated in Figure 2. The difference of PPAR transactivation between RPE cells and 3T3-L1 cells might be related to the fact that the liver tissues and cells express both PPAR-γ1 and PPAR-γ2 isoforms [53], whereas the porcine RPE cells express only the PPAR-γ1 isoform [53, 54]. Further experiments would be required to determine whether PPAR-γ1 and PPAR-γ2 isoform transactivation profiles by norbixin are equivalent.

By competitive binding experiments using radiolabelled ligands, we showed that norbixin is a ligand of PPAR-γ, and of PPAR-α. However, in our experimental conditions, norbixin alone was unable to induce the transactivation of PPAR. We also showed that norbixin did not inhibit the transactivation of PPAR-α and -β/δ isoforms stimulated by high-affinity synthetic agonists (GW9578 and GW0742, respectively), and norbixin only partially inhibited PPAR-γ transactivation induced by low (1 μM), but not higher (10 μM) concentrations of TGZ. By contrast, norbixin inhibited the transactivation of all three isoforms of PPARs induced by A2E. The comparison of the respective affinities for PPARs of the selective PPAR agonists and of A2E versus the affinity of norbixin, considered as a competitor, could explain these apparently contradictory results. Indeed, we showed that A2E binds PPAR-α and PPAR-γ with low affinities (K_Ds_ of 4.1 × 10^−5^ M and 1.4 × 10^−5^ M, respectively). A2E affinity for PPAR-α is similar but slightly lower than norbixin’s affinity for PPAR-α (K_D_ of 4.1 × 10^−5^ M for A2E and 1.5 × 10^−5^ M for norbixin) and the affinity of A2E for PPAR-γ is ten times inferior to the affinity of norbixin for PPAR-γ (KD of 1.4 × 10^−5^ M and 1.0 × 10^−6^ M respectively). Therefore, in theory norbixin is able to displace A2E from PPAR-α and -γ isoforms but is unable to compete with the synthetic high-affinity PPAR-α and β/δ agonists GW9578 and GW0742, whose binding affinities are in the nanomolar range, and only moderately with TGZ, whose binding affinity to PPAR-γ has been reported to be 1 μM [10].

Because PPAR is a permissive NR, we hypothesized that inhibition of A2E-induced PPAR transactivation by norbixin may result not only from the direct binding of norbixin to PPAR, but also by an indirect effect through binding of norbixin to RXRs. We report in the present study that norbixin binds to RXR-α with low affinity but does not induce the transactivation of RXR in our experimental conditions. Moreover, while norbixin does not inhibit the transactivation of RXRs induced by HX630, a high-affinity pan-agonist of RXRs norbixin partially inhibits A2E-induced transactivation of RXRs. This incomplete inhibitory effect could be explained by the respective binding affinities of A2E and norbixin for RXR-α. Indeed, the binding affinity of A2E for RXR-α is 10-fold higher than the binding affinity of norbixin for RXR-α (K_Ds_ of 4.3 × 10^−6^ M and 4.6 × 10^−5^ M, respectively). Therefore, it is expected that norbixin can only partially compete with A2E for RXR-α. In addition, the interactions between A2E and norbixin with RXR-β and -γ were not evaluated here, but should also be taken into account and may partly explain the incomplete inhibition by norbixin of A2E-induced RXR transactivation.

Altogether, our observations suggest that, in the context of porcine RPE cells *in vitro*, norbixin behaves as a low-affinity neutral antagonist of RXRs and PPARs. Moreover, we suggest that the di-apocarotenoid, norbixin, regulates A2E biological activity by antagonizing partially PPAR and RXR. These partial inhibitory capacities seem correlated with the anti-inflammatory effects of norbixin *in vitro* and also potentially explain our previous published results showing that norbixin is protective in various *in vivo* animal models of AMD [23, 24]. More broadly modulation of NRs by carotenoids such as lutein/zeaxanthin or β-carotene may explain their reported beneficial effects in AMD patients by modulating A2E’s effects [48, 49].

### Limitations of the study

The main weakness of our paper is the fact that it is a pure *in vitro* model that does not encompass all the phenomena at play during AMD *in vivo*. In particular, the effect of A2E and norbixin on NRs expressed in other cell types including macrophages, photoreceptor neurons and blood vessels that are important cell types involved in the AMD pathology were not studied. The exact role of A2E in AMD physiopathology remains a subject of debate due to the lack of spatial correlation between A2E concentration and lipofuscin autofluorescence. In addition, degradation products of A2E are possible ligands for NR which can be activated at the nM range. Therefore, despite the fact that we confirmed the purity at 98% of A2E used in the present study, we cannot exclude that the effects that we attribute to A2E are mediated at least partly by other potential degradation products. Nevertheless, it should be emphasized that these A2E degradation products and other NR ligands that may be present in our *in vitro* experiments, are also potentially accumulating in the retina of AMD and STGD patients. Thus, our *in vitro* model could still be representative of molecular mechanisms involved in these pathologies. Another limitation is due to the fact that this study focuses only on PPARs and RXR as we could not obviously study the role of all the NRs (RAR for instance) that could be at play following RPE exposure with A2E. Complementary experiments are actually performed to evaluate more precisely the role of RAR transactivation induced by A2E in the modulation of inflammation.

In conclusion, we show that A2E induces the transactivation of RXR and PPAR and that an important part of the biological effects of A2E may be mediated through activation of these NRs. These observations bring new insights of the physiopathology of AMD. Moreover, we demonstrate that norbixin partially inhibits A2E-induced RXR transactivation and behaves as a pan-inhibitor of PPAR transactivation by A2E. Finally, norbixin modulates the expression of molecules involved in angiogenesis and inflammation stimulated by A2E and that are critical for AMD evolution. Consequently, our study suggests that modulation of NR by norbixin or related molecules could open new avenues for the treatment of AMD.

## MATERIALS AND METHODS

### Reagents/Chemicals

All usual chemicals, primers, and HX531 were from Sigma (St. Louis, MO, USA). Reagents for cell culture, transfection and quantitative RT-PCR were from Thermo Fisher Scientific (Waltham, MA, USA). RNA extraction NucleoSpin^®^ RNA kit was from Macherey Nagel (Düren, Germany). ECL prime and PVDF membrane were from Amersham GE Healthcare (Buckinghamshire, UK). Primary antibodies against the following proteins were used: GAPDH (Santa Cruz Biotechnology, Inc, Dallas, TX, USA); pAKT, AKT, pERK and ERK (Cell Signaling, Danvers, MA, USA). Secondary antibodies were from Jackson ImmunoResearch (Cambridgeshire, UK). GW9578 was from Cayman Chemical Company (Ann Arbor, MI, USA) and GW0742, TGZ, T007907, GSK3787, MK886 and HX630 were purchased from TOCRIS (Bristol, UK). Cignal Pathway Reporter Assay Kits were from QIAGEN (Frederick, MD, USA). The Dual-Luciferase Reporter Assay System was purchased from Promega (Madison, WI, USA). pcDNA 3.1 (+)-PPARα and pcDNA 3.1 (+)-PPARβ/δ (pig sequence) were purchased from Genscript (Piscataway, NJ, USA). pCMV6-XL4-PPARγ (human sequence) was purchased from Origene (Rockville, MD, USA).

### Synthesis of norbixin

9’-*cis*-norbixin was prepared from 9’-*cis*-bixin (AICABIX P, purity 92%) purchased from Aica-Color (Cusco, Peru) upon alkaline hydrolysis as previously described [23]. Purity at 97% was determined by HPLC.

### Synthesis of A2E

A2E was synthesized by Orga-link (Magny-Les-Hameaux, France) as described before [23]. A2E had a 98% purity as determined by the provider and was confirmed by HPLC internally for each new batch used in our experiments.

### Binding studies to RXR-α and PPAR-α and γ

Binding studies of A2E and norbixin to RXR-α and PPAR-α and γ were performed *in vitro* by an external laboratory (Eurofins Cerep, Celle L’Evescault, France) through competition experiments between A2E or norbixin and [^3^H]9-*cis*-retinoic acid as the natural ligand of RXR-α, [^3^H]GW7647 an agonist of PPAR-α and [^3^H]Rosiglitazone an agonist of PPAR-γ.

### *In vitro* model of RPE cell culture and treatments

Pig eyes were obtained from a local slaughterhouse and transported to the laboratory in ice-cold Ringer solution. After removal of the anterior segment of the eye, the vitreous and neural retina was separated from the RPE and removed. The eyecup was washed twice with phosphate buffer saline (PBS), filled with trypsin (0.25% in PBS) and incubated at 37°C for 1.5 h. RPE cells were harvested by gently pipetting, centrifuged to remove trypsin and re-suspended in Dulbecco’s Modified Eagle Medium (DMEM) supplemented with 20% (v/v) foetal-calf serum (DMEM20%FCS) and 0.1% gentamycin. Cells were seeded into 60 mm diameter Petri dishes, cultured in an atmosphere of 5% CO2/95% air at 37°C, and supplied with fresh medium after 24 hours and 4 days *in vitro*. For experiments aimed at measuring mRNA or protein expression in RPE, cells were trypsinized after one week in culture and transferred to 24-well or 6-well plates at a density of 1.5 × 10^5^ cells/cm^2^ in DMEM2%FCS. Norbixin or agonists were added to the medium 24 h after seeding, and antagonists were added to the medium 24 h later. A2E treatment was performed during 19 h before cellular sample preparation. All the drugs used in these experiments were prepared as stock solutions in DMSO.

### PPAR, RXR, AP-1 and NF-κB transactivation assays

After one week in culture, cells were trypsinized and transferred to a 96-well plate at a density of 6 × 10^4^ cells/cm^2^ in DMEM2%FCS. The next day, cells were transfected using the Cignal Reporter Assay Kits for PPAR, RXR, AP-1 and NF-κB, according to the manufacturer’s specifications. To measure the specific activation of PPAR isoforms, cells were also co-transfected with PPAR-α, PPAR-β/δ or PPAR-γ expression vectors. Transfection was performed with Lipofectamine and Plus Reagent in serum-free medium. 3 h after the transfection, the medium was replaced and treatments with the molecules to assay were performed. Luciferase activity was measured the next day using the Dual-Luciferase Reporter Assay System. The measurements were performed with a luminometer (Infinite M1000 from Tecan, Mannedorf, Switzerland). Firefly: Renilla activity ratios were calculated for each condition and ratios from transcription factor-responsive reporter transfections were divided by ratios from negative control transfections to obtain relative luciferase unit, as described by the manufacturer. At least 3 independent transfections were performed in triplicate for each condition.

### Quantitative RT-PCR

Total RNA was extracted using the NucleoSpin^®^ RNA kit according to manufacturer’s instructions. RT of 500 ng of RNA was performed using the SuperScript III Reverse Transcriptase following the manufacturer’s instructions. Five ng of cDNA were amplified using the SYBR GREEN real-time PCR method. PCR primers for target genes and housekeeping gene GAPDH were designed using Primer3Plus Bioinformatic software (Table 2). The RT-PCR using the StepOne Plus (Life Technologies, Carlsbad, CA, USA) consisted of incubation at 50°C for 5 min followed by 40 cycles of 95°C for 15 s and of 60°C for 1 min. The reaction was completed by a melt curve stage at 95°C for 15 s, 60°C for 1 min, 95°C for 15 s and a final step at 60°C for 15 s. The relative mRNA expression was calculated using the comparative threshold method (Ct-method) with GAPDH for normalization. All experimental conditions were processed in triplicate and each experiment was done at least 3 times.

**Table 2:** Probes used for mRNA quantification by RT-QPCR.

**Gene**	**Sequence**	
**GAPDH**	**F**	GCTGCTTTTAACTCTGGCAA
	**R**	CCACAACATACGTAGCACCA
**IL-6**	**F**	CGGATGCTTCCAATCTGGGT
	**R**	CACAGCCTCGACATTTCCCT
**IL-18**	**F**	ACTTTACTTTGTAGCTGAAAACGATG
	**R**	TTTAGGTTCAAGCTTGCCAAA
**IL-8**	**F**	GGCAGTTTTCCTGCTTTCT
	**R**	CAGTGGGGTCCACTCTCAAT
**VEGF A**	**F**	GTCTGGAGTGTGTGCCCA
	**R**	GTGCTGTAGGAAGCTCATC
**MMP9**	**F**	GGCAGCTGGCAGAGGAATATC
	**R**	GAAGCTTTAGAGCCGGTTCCA
**CCL2**	**F**	TGCCCAGCCAGATGCAATTA
	**R**	TGCTGCTGGTGACTCTTCTG

### Protein analysis

RPE samples were lysed in 20 mM Tris-HCl, 150 mM NaCl, 1 mM EDTA, 1% NP-40, pH 7.5 buffer containing a cocktail of protease and phosphatase inhibitors. Equal amounts of protein were resolved by 12% SDS polyacrylamide gel electrophoresis and electro-transferred onto a PVDF membrane using a standard protocol. The membranes were blocked with 5% milk or 10% BSA for 1 h, followed by incubation with a primary antibody overnight at 4°C. Subsequently the membranes were incubated with the corresponding horseradish peroxidase-conjugated secondary antibodies for 1 h. The signal was developed using enhanced chemiluminescence reagents (ECL) prime detection kit, quantified by densitometry using Bio1D (Vilber Lourmat, Germany) and normalized by GAPDH levels. Each experiment was done at least 3 times.

### Statistical analyses

For statistical analyses one-way ANOVA followed by Dunnett’s tests were performed using Prism 7 (GraphPad Software, La Jolla, CA, USA).
